# Validation of the Paediatric Nijmegen Questionnaire in Sweden: A Self‐Screening Tool for Dysfunctional Breathing

**DOI:** 10.1111/crj.70221

**Published:** 2026-08-06

**Authors:** Eleni Theofanidou‐Axelsson, Malin Nygren‐Bonnier, Björn Nordlund, Henrik Ljungberg, Helene Alexanderson

**Affiliations:** ^1^ Department of Medicine Solna, Division of Rheumatology Karolinska Institute Stockholm Sweden; ^2^ Women's Health and Allied Health Professionals Theme, Medical Unit Allied health professionals Karolinska University Hospital Stockholm Sweden; ^3^ Department of Neurobiology, Care Sciences and Society Karolinska Institute Stockholm Sweden; ^4^ Astrid Lindgren Children's Hospital, Lung and Allergy Unit Karolinska University Hospital Stockholm Sweden; ^5^ Department of Women's and Children's Health Karolinska Institute Stockholm Sweden

**Keywords:** adolescent, asthma, child, dysfunctional breathing, Nijmegen Questionnaire

## Abstract

**Background:**

The Nijmegen Questionnaire (NQ) is used to identify Dysfunctional Breathing (DB) in individuals with asthma. However, it has not been validated for use in children and adolescents.

**Objective:**

This study aimed to validate a Swedish NQ for this population.

**Methods:**

Participants aged 10–17 years with asthma were included (*n* = 124). In Cohort I (*n* = 22), audiotaped semi‐structured interviews were conducted to adapt and refine the questionnaire analyzed by qualitative content analysis, resulting in NQ version 1. Forty‐eight participants (11 from Cohort I, 37 new) then completed version 1 to assess internal redundancy, consistency (Spearman's Rho, Cronbach's alpha) and item clarity, leading to version 2. Cohort II (*n* = 60 new) completed NQ version 2 alongside the Paediatric Asthma Quality of Life Questionnaire (PAQLQ) and Childhood/Adult Asthma Control Test (cACT/ACT) to assess construct validity. Cohort III (*n* = 54, 48 from Cohort II, 6 new) completed NQ version 2 again after 2 weeks for test–retest reliability.

**Results:**

Content analysis prompted revisions for clarity and relevance. Internal redundancy ranged from r_s_ = 0.00–0.69 and internal consistency was 0.85 (Cronbach's alpha). The NQ correlated moderately with cACT/ACT (r_s_ = −0.54) and PAQLQ subscales Symptoms, Activity limitation and Emotional function, r_s_ −0.52, −0.48, −0.39 (CI 0.32;0.70, 0.24;0.66, 0.14;0.60) respectively. Test–retest reliability was 0.86 (Kw) (*p* < 0.0001).

**Conclusion:**

The Swedish Paediatric NQ seems to be a valid and reliable tool for identifying DB in children and adolescents with asthma.

## 1 Introduction

Asthma is the most common respiratory disease in children and one of the most common reasons for emergency hospital visits [1]. In asthma, chronic inflammation in the airways leads to, among other things, swelling of mucous membranes, increased mucus production and contraction of airway muscles. Asthma is a heterogeneous disease that varies between individuals over time caused by allergic or non‐allergic factors such as physical activity. Common symptoms are coughing, wheezing, chest pressure, difficulty breathing or shortness of breath. Exercise‐induced asthma is very common in children and adolescents and approximately 80% of children with untreated or poorly controlled asthma also have exertion‐related complaints. Exercise‐induced asthma is very common in children with other forms of asthma and has been shown to be strongly associated with low quality of life in all children and adolescents and their caregivers [2].

In adults with asthma, a high‐costal breathing pattern is characterized by reliance on the upper chest and accessory respiratory muscles rather than the diaphragm and is well‐documented [3, 4]. This type of high‐costal breathing is considered dysfunctional, as it leads to inefficient ventilation and overuse of accessory muscles, often resulting in muscle tension, tightness, as well as respiratory symptoms such as shortness of breath, chest pressure and chest pain. These symptoms can further contribute to non‐respiratory issues including anxiety, dizziness and fatigue, all of which negatively impact quality of life [4, 5].

Current understanding of breathing pattern changes in children and adolescents with asthma is still limited. However, evidence shows that many in this group also display similar dysfunctional breathing patterns. There is no consensus definition of dysfunctional breathing (DB); however, it generally involves minimal diaphragmatic engagement and increased activity of accessory muscles [3, 4, 6, 7]. Dysfunctional breathing is also associated with poor asthma control as patients might not respond to asthma medication and is frequently misclassified as ‘difficult‐to‐treat asthma.’ DB can also present in the absence of asthma; however, most patients with chronic DB also have an asthma diagnosis [8].

DB is often under‐diagnosed and its prevalence is difficult to determine due to the absence of gold standard diagnostic criteria [4, 9, 10, 11]. The Nijmegen Questionnaire (NQ) was introduced in 1985 to assess symptoms related to DB [12] and it was first validated for adults with asthma in a Greek population [13]. The NQ is used in studies on adults with chronic obstructive pulmonary disease (COPD) and asthma [10, 14, 15] as well as in children [16] with asthma in Denmark to assess DB. It demonstrated sensitivity and specificity in measuring subjective symptoms as a marker for DB in adults [9, 17]. Many adult patients with asthma have an elevated NQ score corresponding with poor asthma control [4, 9].

The NQ is available in a Swedish version; however, it is not published and is used in resources such as the website of the Asthma and Allergy Association, the Swedish patient organization. However, to our knowledge, the NQ has not previously been validated for use in children and adolescents in any language or country. In addition, there is no other available validated instrument to measure symptoms related to DB in young people with asthma. Children differ from adults in their physiological, psychological and developmental characteristics, as well as in how they perceive, report and interpret respiratory symptoms. Therefore, our study aimed to validate the NQ for a paediatric Swedish population with asthma in terms of content validity, construct validity and test–retest reliability.

## 2 Materials and Methods

### 2.1 Participants

**FIGURE 1:**
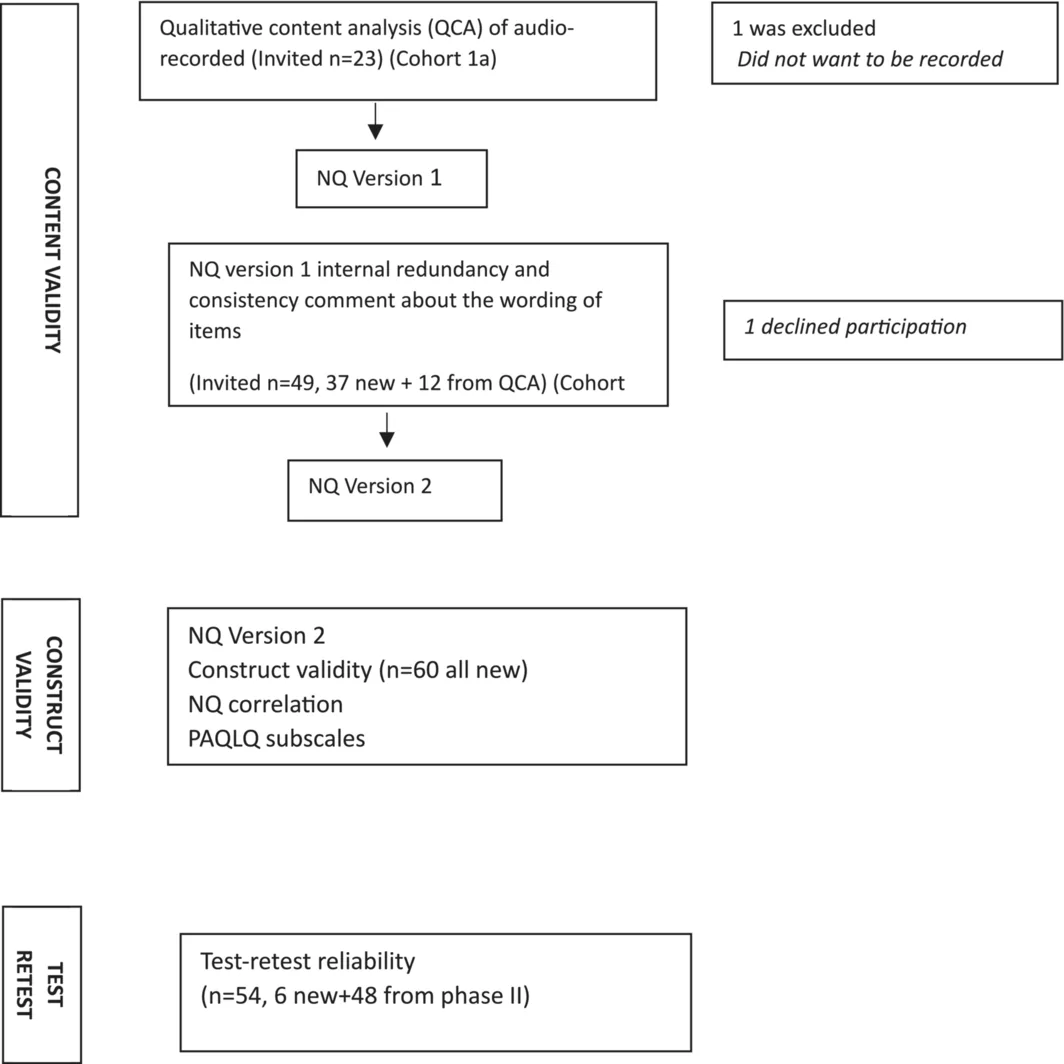
Flowchart of the study design and inclusion/exclusion. A total of 124 participants were included.

A mixed‐methods design conducted over three steps was used, including three cohorts. All participants were recruited from Astrid Lindgren Children's Hospital, Children's (Pulmonary) Asthma Allergy clinic and Karolinska University Hospital, Stockholm. Altogether, 124 individual participants were included in the study (Figure 1).

#### 2.1.1 Cohort Ia and Ib Content Validity

In cohort 1a, participants were strategically identified from outpatients at the children's asthma and allergy clinic to ensure representation across ages (10–17 years), sex and various degree of asthma and DB (*n* = 22). Inclusion criteria: age between 10 and 17 years, diagnosis of asthma with or without dysfunctional breathing, ability to read and understand the survey questions. Exclusion criteria: No diagnosis of asthma or dysfunctional breathing, inability to comprehend or complete a questionnaire in Swedish. Participants first completed the original Swedish version of NQ and provided feedback on its content through individual audiotaped interviews (Cohort Ia). The original NQ was revised according to the feedback (cohort Ia) leading to the Paediatric NQ version 1. In the next step, cohort Ib consisted of 48 participants, (11 from cohort 1a with 37 additional participants). They were recruited to complete the revised version of the NQ and again comment on content which led to Paediatric NQ version 2.

#### 2.1.2 Cohort II, Construct Validity

Cohort II consisted of 60 new participants (none participated in cohorts 1a and 1b). Participants were included during the fall of 2023, using the same inclusion and exclusion criteria as in cohort 1a and b.

#### 2.1.3 Cohort III, Test–Retest Reliability

Cohort 3 consisted of 54 participants (48 from phase 2 along with an additional 6 participants with stable asthma defined as a score of > 20 of Childhood Asthma Control Test [cACT/ACT]). Participants were included during winter of 2023. Demographic data on all participants are presented in Table 1.

**TABLE 1:** Demographic characteristics for all participants (*n* = 124) in cohorts I‐III in various parts of the study, age range 10–17.

	Cohort I (*n* = 48)	Cohort II (*n* = 60)	Cohort III (*n* = 54)
	Content validity	Construct validity	Test–retest
Male/Female, n	22/26	40/20	35/19
Age, years, median (range)	13 (10–17)	13 (10–17)	14 (10–17)
Type of asthma			
Asthma, *n* (%)	7 (15)	18 (30)	13 (24)
Mixed asthma, *n* (%)	17 (35)	27 (45)	26 (48)
Atopic asthma, *n* (%)	13 (27)	8 (13)	9 (17)
Non atopic asthma	8 (17)	7 (12)	6 (11)
Children with DB, *n* (%)	19 (40)	25 (40)	16 (29)
Male/Female, *n* (%)	2/17 (4/35)	13/12 (52/48)	11/5 (69/31)
Girl's median with DB (range)	14 (11–17)	13.5 (12–17)	13,8 (12–17)
Boys median with DB (range)	14 (12–16)	13 (12–16)	13.6 (12–16)
Medication			
Bufomix/Buventol/airomir (*n*/%)	41 (85)	45 (75)	40 (74)
Giona	11 (23)	5 (8)	4 (7)
Flutiform	4 (8)	5 (8)	3 (5)
Singulair	4 (8)	2 (3)	3(5)
Other	6 (10)	3 (5)	4 (7)
PAQLQ median, (range)	NA	139 (96–161)	NA
cACT. Median (range)	NA	22 (8–25)	22 (20–25)
NQ median, (range)	23 (14–37)	16 (1–49)	11 (3–33)

Abbreviations: cACT: childhood Asthma Control Test, DB: dysfunctional breathing, NQ: Nijmegen Questionnaire, PAQLQ: Paediatric Asthma Quality of Life Questionnaire.

The sample sizes for the three cohorts were determined based on methodological recommendations for psychometric validation studies, together with feasibility and participant availability. Cohort I (*n* = 48) was considered adequate for content validity assessment, Cohort II (*n* = 60) for construct validity testing and detection of moderate effect sizes and Cohort III (*n* = 54) for test–retest reliability analyses. Although no formal a priori power calculation was performed for all analyses, the sample sizes were considered consistent with commonly accepted standards for instrument validation studies.

### 2.2 Data Collection

The original NQ comprises 16 items aimed at assessing symptoms of dysfunctional breathing (DB). Seven of these are associated with respiratory symptoms, four with excessive ventilation and five with central nervous system symptoms linked to hypocapnia and central tetany. Each item is rated on a five‐point ordinal scale, from 1 (never) to 5 (very frequently) [12]. The NQ has been extensively validated in adults with asthma and shows good correlation with objective measures of DB [13] It demonstrates high sensitivity (91%) and specificity (95%) for identifying healthy individuals with DB. Reported cut‐off scores for DB range between 20 and 23 [13].

The Paediatric Asthma Quality of Life Questionnaire (PAQLQ) measures the impact of asthma on children and adolescents aged 6 to 17 by evaluating three areas: asthma symptoms, activity limitations and emotional function [18, 19]. A Likert scale is used to generate an overall quality of life score ranging from 23–161 (161 indicates very good QoL). It The PAQLQ is validated as to construct validity, internal consistency and test–retest/reproducibility as well as sensitivity to change in children with asthma [18, 19] and is widely used in clinical practice and research to understand the patient's perspective.

The Asthma Control Test (ACT) for adults and children aged 12+ and the Childhood ACT (cACT) are tools used to assess asthma control, guiding treatment decisions [20, 21]. The ACT includes 5 questions on symptoms, activity impact, reliever medication use and overall asthma control over the past 4 weeks. Scores range from 5 to 25, with a score of 20 or higher indicating well‐controlled asthma, with lower scores signalling the need for treatment adjustments. The c‐ACT is valid for children aged 4–11 years, relying on parental assessment and includes four questions about symptoms, nighttime awakenings, overall control and reliever use. The scoring is similar to the ACT, with a total score ranging from 4 to 27, where a score below 20 indicates poorly controlled asthma. Both tools are simple, evidence‐based and effective for assessing asthma and guiding treatment in both adults and children. The c‐ACT and ACT are commonly used questionnaires for assessing asthma control [22].

### 2.3 Procedure

#### 2.3.1 Cohort I: Content Validity

Content validity was assessed using qualitative methods through individual interviews with participants and, for those under 15 years old, their parents, cohort Ia. Participants completed the original NQ and provided feedback on its content, clarity and relevance of questions and answer options. The interviews were conducted in person, lasting a median of 15 min (range 10–30 min). A semi‐structured interview guide with open‐ended questions focusing on the relevance and understanding of items was administered to all participants to ensure methodological uniformity while allowing for exploration of comprehension, wording clarity and conceptual relevance in a child context (Table 2). All interviews were audio‐recorded and transcribed verbatim. Based on qualitative analysis, instruction and wording were refined leading to the development of a revised NQ (version 1). This version was then completed by a larger group of participants (Cohort Ib) who also provided feedback on content and clarity. Interviews were conducted until data saturation was achieved, defined as the point at which no new issues related to comprehension difficulties, ambiguous terminology, or conceptual misinterpretations were identified across consecutive interviews. Saturation was further supported by the recurrence of similar patterns of misunderstanding and repeated identification of specific problematic terms. The revised questionnaire was analyzed for internal redundancy and internal consistency, resulting in the final Paediatric NQ version.

**TABLE 2:** Semi‐structured interview guide.

1. Do you understand the questions, is the language easy to understand? 2. Are there any important areas that are not included/is missing? 3. Are the questions relevant to you? 4. Is there anything else you or your parent would like to add?

#### 2.3.2 Cohort II: Construct Validity

For construct validity purposes, participants in cohort II were invited to complete the final Paediatric NQ version, the PAQLQ [18] and the ACT/c‐ACT. Total score of Paediatric NQ was then correlated with PAQLQ and ACT/cACT. All patients were invited to participate and completed the questionnaires during a clinic visit, except for two participants who completed the questionnaires at home.

Our a priori hypothesis was that the NQ total score would correlate best to cACT/ACT and PAQLQ subscale asthma symptoms (moderate correlation), with lower correlations for PAQLQ subscales activity limitations and emotional function.

#### 2.3.3 Cohort III, Test–Retest Reliability

All participants in cohort II with stable asthma (> 20 points on the ACT/cACT in the last 3 months) completed the Paediatric NQ a second time (retest) at home 2 weeks later and returned it to the clinic by mail.

### 2.4 Ethical Considerations

The study was approved by the Ethics Review Authority in Sweden (diary number 2021‐06559‐01). Written informed consent was obtained from all participants before data collection began. Participants aged 15 years or older provided their own consent, whereas consent for children under 15 years was obtained from both legal guardians and the child. Permission to gather and store patient information was granted in accordance with ethical guidelines.

### 2.5 Analysis of Qualitative Data

Individual interviews were transcribed verbatim and reviewed multiple times by the first and senior authors, ETA and HA, to ensure a comprehensive understanding. Key meaning‐bearing units were identified, condensed and coded under their specific items. Responses were mainly focused on how items were formulated and how response alternatives were constructed. Based on participants' feedback, the NQ instructions and several items were revised to improve clarity and comprehension. An iterative process of cognitive evaluation and item refinement was used throughout the adaptation phase. Feedback from participants regarding unclear wording, age‐inappropriate expressions and difficulties in interpreting items was systematically documented and analyzed after each interview round. Revisions to item wording and structure were implemented in a stepwise manner based on emerging findings, ensuring progressive improvement of clarity and age‐appropriateness.

To strengthen methodological rigour and reduce individual interpretive bias, all proposed modifications were critically reviewed in multidisciplinary consensus meetings involving the research team (ETA, MNB and HA). Final decisions regarding item adaptation were made only after consensus was achieved. This collaborative process ensured semantic clarity, conceptual equivalence and developmental appropriateness of the adapted instrument for the target paediatric population. The analysis process is illustrated in Table 3.

**TABLE 3:** Example of the qualitative content analysis process.

Meaning unit	Condensation	Code
*‘IT should have been stated on the questionnaire! That it is in connection with breathing difficulties. It is NOWHERE mentioned!’* (Male‐ID:13).	It should state it's about breathing issues—nowhere is that mentioned	Lack of instructions in the questionnaire.
*‘What do you mean by daze?. A feeling of being far away?* I do not understand’ (Male‐ID:20).	A state of confusion or not thinking clearly, like your mind is far away	Difficulty understanding sentences and words
*‘Do you mean these symptoms during activity?’ ‘The sentences and words are quite easy to understand, it's the context around it …that is difficult to understand’ (Male‐ID:13)*.	Do you mean these symptoms during activity? The words are easy, but the context is hard to understand	Unclear in what context the symptoms are supposed to occur
*‘Add a number for each question would make it easier, specify for younger children’ (Female‐ID:18, parent suggests)*	Number the questions to simplify for younger children	Number each item

### 2.6 Statistical Analysis

Statistical analysis was conducted using STATA/BE version 18 (StataCorp). Demographic data are presented as median (range). Since all outcome measures produce ordinal data, non‐parametric statistical methods were applied. Internal redundancy was assessed using Spearman's rho, whereas internal consistency was evaluated using Cronbach's alpha. Low internal consistency was defined as a value below 0.60 and internal redundancy was considered present when Spearman's rho exceeded 0.90. Around 60 patients are needed to reach stable correlation coefficients [23]. Construct validity was also analyzed using Spearman's rho. Correlation strength was categorized based on Munro's classification, where values between 0 and 0.29 indicated no to very low correlation, 0.30 to 0.49 indicated low to moderate correlation, 0.50 to 0.69 indicated moderate to high correlation, 0.70 to 0.89 indicated high correlation and values above 0.90 indicated very high correlation [23].

Test–retest agreement was evaluated using weighted kappa. The significance level was set at *p* < 0.05. Weighted kappa values between 0 and 0.20 were considered to indicate no or low agreement, values from 0.21 to 0.40 indicated fair agreement, 0.41 to 0.60 indicated moderate agreement, 0.61 to 0.80 indicated substantial agreement and values from 0.81 to 1.00 indicated almost perfect agreement [24].

## 3 Results

All together 124 children and adolescents aged 10–17 years participated in the study. Complete demographic data are presented in Table 1.

Children and adolescents in cohort 1a described experiencing shortness of breath during asthma attacks, though most did not consciously think about their breathing or breathing pattern. Nine participants who all scored high on the NQ scale (> 20 points, *n* = 22) identified items as relevant and described their symptoms as ‘difficulty getting air into the lungs’ or ‘not getting enough air’. Example of the analysis process is represented in Table 3. Eight of the participants scoring lower on the NQ expressed that their scores would have been higher if asked before undergoing the hypo‐sensibility treatment.

The participants suggested modifying certain difficult words in the NQ to improve understanding. The questionnaire did not include instructions clarifying whether symptoms of breathing problems should be considered in a specific context. As a result, 18/22 participants were unsure whether the items referred to breathing difficulties during physical activity or at rest. This was clarified as ‘Circle the number that best describes how often you are bothered by the following symptoms associated with respiratory problems, regardless of whether you are resting or physically active’. Six of 22 participants found item 5 unclear, specifically whether ‘increased heart palpitations’ referred only to physical activity, such as running, or if it was related to breathing problems in general. Additionally, 20/22 participants found the term ‘Daze/Light headedness’ difficult to understand. Despite modifications in NQ version 1, 18/22 participants still struggled with this term, leading to its replacement with *‘confused.’* Ten of 22 participants found the word ‘dizzy’ difficult to understand. Furthermore, the original questionnaire did not number the items, making it challenging to discuss specific questions during the interviews. To improve clarity, each item was numbered in the revised version. All revisions leading to the NQ version 2 are presented in Table 4.

**TABLE 4:** The final version of Paediatric Nijmegen Questionnaire translated from Swedish to English. Bolded text highlights revisions from the adult NQ.

	Never	Seldom	Sometimes	Often	Very often
**1. Tension** in the body	0	1	2	3	4
**2.** Difficult to get air	0	1	2	3	4
**3.** Need for **extra** fast and/or deep breaths	0	1	2	3	4
**4.** Difficulty breathing deeply (get air into the lungs)	0	1	2	3	4
**5.** Palpitations/beating heart (**the heart beats harder**)	0	1	2	3	4
**6.** Cold hands or feet	0	1	2	3	4
**7.** Feeling anxious or anxious	0	1	2	3	4
**8.** Pain/aches or tingling in the chest	0	1	2	3	4
**9.** Feeling **dizzy**	0	1	2	3	4
**10. Tight chest**	0	1	2	3	4
**11.** Tingling or numbness (**loss of sensation**) in the fingers	0	1	2	3	4
**12**. Blurred vission	0	1	2	3	4
**13**.**Feeling confused**	0	1	2	3	4
**14**. Stiffness in fingers or arms	0	1	2	3	4
**15. Feeling bloated** in the stomach	0	1	2	3	4
**16. Feeling stiff/tight** around the mouth	0	1	2	3	4

*Note:* Circle the number that best describes how often you are bothered by the following symptoms associated with respiratory problems, regardless of whether you are resting or physically active.

In the analysis of internal redundancy, Spearman's rho ranged from r_s_0.00 to r_s_0.69, indicating that no items were redundant. Cronbach's alpha coefficient was 0.86, confirming that all items contributed sufficiently to the total score. As a result, no items were removed.

Our a priori hypothesis regarding construct validity was confirmed. As expected, the strongest correlation was between NQ and ACT/cACT (r_s_ = −0.54, 95% CI: 0.33;0.70). There was a similar correlation between NQ and the PAQLQ subscale Asthma symptoms (r_s_ = −0.52, 95% CI: 0.31;0.68) with lower correlations between NQ and the PAQLQ subscale Activity limitation (r_s_ = −0.48, 95% CI: 0.26;0.65) and Emotional function (r_s_ = −0.39, 95% CI: 0.15;0.59).

The weighted kappa coefficient between test and retest was K_W_0.86 (*p* < 0.0001), indicating excellent agreement. Total score of test and retest, median and range is illustrated in Table 5.

**TABLE 5:** Test–retest reliability of single items and total score of the Paediatric NQ.

Paediatric Nijmegen Questionnaire items	TEST md (range)	RETEST md (range)	Weighted kappa
Q1	1 (0–4)	0 (0–3)	
Q2	2 (0–4)	2 (0–4)	
Q3	2 (0–4)	2 (0–4)	
Q4	2 (0–4)	1 (0–4)	
Q5	1 (0–4)	1 (0–4)	
Q6	0 (0–4)	0 (0–4)	
Q7	0 (0–4)	0 (0–4)	
Q8	1 (0–4)	1 (0–3)	
Q9	0 (0–4)	0 (0–3)	
Q10	1 (0–4)	1 (0–4)	
Q11	0 (0–2)	0 (0–3)	
Q12	0 (0–2)	0 (0–2)	
Q13	0 (0–3)	0 (0–3)	
Q14	0 (0–3)	0 (0–2)	
Q15	0 (0–2)	0 (0–3)	
Q16	0 (0–4)	0 (0–3)	
Total score	12 (1–49)	11 (1–33)	0.86

## 4 Discussion

This study aimed to validate the Swedish translated NQ for use in Swedish speaking children and adolescents. The validation process included content and construct validity as well as test–retest reliability. Several items and instructions required revision to enhance clarity and understanding. All items of the revised NQ contributed meaningfully to the total score, with no redundancies. Furthermore, the NQ showed a moderate correlation with the cACT/ACT and slightly lower correlations with quality‐of‐life measures. The total score demonstrated excellent test–retest reliability. The validation process led to the first Paediatric NQ (PNQ).

Our qualitative analysis highlighted the need to refine item wording and instructions to improve clarity. This underscores the importance of involving patients in the development and validation of self‐reported scales to ensure they are understandable and meaningful. However, to our knowledge, the development and validation of the original adult NQ did not include the patient perspective of DB [12, 25].

The PNQ, enhanced for understanding and clarity, demonstrated high internal consistency, with a Cronbach's alpha of 0.85. This is comparable to several other translated versions of the adult NQ, which range from 0.70 to 0.92 [13, 26, 27, 28]. This finding supports the internal consistency reliability of the PNQ and confirms its successful validation and adaptation for children and adolescents in Sweden. Additionally, our results indicated that no items exhibited redundancy. As far as we know, internal redundancy has not been assessed in other translated versions of the adult NQ [13, 26, 27, 28].

The final version of the Paediatric NQ showed a moderate correlation with the ACT/cACT, which was expected since symptoms of dysfunctional breathing can overlap with asthma symptoms [25]. Similarly, the Greek and Turkish adult versions of the NQ also demonstrated moderate correlations with the ACT [13, 27]. Furthermore, the final PNQ had moderate correlations with the PAQLQ subscales for asthma symptoms and activity limitations, aligning with the hypothesis that DB is associated with asthma control and symptoms and that DB also negatively impacts daily activities [8]. Our results highlight the need for the PNQ to accurately capture symptoms related to DB in children and adolescents with asthma.

Weighted kappa coefficient between test and retest of total score of the Paediatric NQ was 0.86 indicating almost perfect agreement over 2 weeks, which was convergent to the adult Persian version (0.82) [26] and slightly higher than test–retest reliability of the adult Turkish version [27]. The test–retest reliability of the adult Thai version was evaluated over only 2 days, which may be too short, increasing the risk of participants remembering their initial responses [28]. In contrast, we conducted test–retest evaluation over 2 weeks to minimize memory bias. Although this longer interval of 2 weeks introduces the potential risk of capturing fluctuations in DB symptoms, we could demonstrate an excellent test–retest reliability of the Paediatric NQ.

There are several ways to assess DB. The Respiratory Movement Respiratory Instrument (RMMI) can objectively assess breathing patterns in different body positions in adults [29]. However, this instrument requires training and is not available broadly and has not been validated for use in young people. In clinical practice, progressive exercise testing is commonly used to detect DB during physical exertion. However, there is limited data on its sensitivity and specificity for detecting DB, especially in asthma patients. In addition, clinicians use a variety of manual outcome measures to assess DB, for example, Hi‐Lo breathing, where the clinician places hands on upper chest and stomach to appraise breathing pattern [30]. These tests require basictraining affecting feasibility and none of them are validated for use in young people [29, 30, 31]. There is also an available self‐reported tool to assess breathing patterns, the Self‐Evaluation of Breathing Questionnaire (SEBQ) [31] and an observational assessment the Total Faulty Breathing Scale (TFBS) [31], however, these have not been validated in adults or children with asthma. The Breathing Pattern Assessment Tool (BPAT) [9, 31] is a clinically validated diagnostic tool for dysfunctional breathing (DB) in patients with difficult asthma. However, there are no published data on its use in children with all types of asthma. The Paediatric NQ, validated in this study, has low patient and assessor burden which enhances its feasibility in clinical practice and research. Vahlkvist et al. [16] recommend routine DB screening in difficult‐to‐treat paediatric asthma to prevent overtreatment and improve symptom management. A high score on the PNQ warrants further investigation by a physical therapist specialist in pulmonary medicine.

This study has some limitations. We were unable to include patients with severe asthma or those experiencing an ongoing asthma attack, as all participants were recruited during outpatient visits in one specialist centre. However, participants in cohort 1a were selected strategically to represent gender, all ages between 10–17 years and severity of DB, which strengthens generalizability in the paediatric population. However, our findings cannot be generalized to young people with severe asthma or to young patients in other countries. Unfortunately, we were unable to validate the NPQ against objective measures such as spirometry. Eleven participants from phase 1a were also included in cohort 1b as we were interested in their perception of the revised NQ compared to the original. Participants were selected based on availability and willingness to participate further, not based on questionnaire responses or performance. We acknowledge that prior exposure to the questionnaire may have introduced familiarity‐related bias.

The original NQ scoring properties, including cut‐off values, sensitivity and specificity, were not assessed in this study. Evaluation of the diagnostic accuracy of the adapted PNQ, including sensitivity and specificity, is ongoing within a randomized controlled trial. Despite these limitations, this is the first study to formally validate the PNQ in any paediatric population with asthma and DB.

In conclusion, our results indicate that the Paediatric Nijmegen Questionnaire demonstrates satisfactory content validity, construct validity and excellent test–retest reliability in children and adolescents with asthma. The Paediatric NQ can enhance the detection of dysfunctional breathing, helping to identify patients who may benefit from treatments beyond pharmacological interventions.

## Author Contributions

**Eleni Theofanidou‐Axelson:** conceptualization, data curation, formal analysis, funding acquisition, investigation, methodology, project administration, validation, writing original draft, writing – review and editing. **Malin Nygren Bonnier:** conceptualization, data curation, funding acquisition, methodology, validation, supervision, writing – review and editing. **Björn Nordlund:** conceptualization, data curation, funding acquisition, supervision, writing – review and editing. **Henrik Ljungberg:** conceptualization, data curation, funding acquisition, supervision, writing – review and editing. **Helene Alexanderson:** conceptualization, data curation, formal analysis, funding acquisition, methodology, project administration, resources, supervision, validation, supervision, writing – review and editing.

## Funding

This work has been supported by grants provided by Promobilia Foundation, Asthma & Allergy Foundation, Jerring Foundation, Anna Lisa and Arne Gustafssons Foundation, Axel Tielmans Memorial Foundation (Crown Princess Loviisa's Association for Children's Health Care) and Mats Klebergs Foundation.

## Ethics Statement

The study was approved by the Swedish Ethical Review Authority (Dnr 2021‐06559‐01). Written informed consent was obtained from all participants before enrolment. Participants aged 15 years or older provided their own written informed consent. For participants younger than 15 years, written informed consent was obtained from both the child and their legal guardians.

## Conflicts of Interest

The authors declare no conflicts of interest.

## Data Availability

The data that support the findings of this study are available on request from the corresponding author. The data are not publicly available due to privacy or ethical restrictions.
